# The Prognostic Value of the Expression of *SMC4* mRNA in Breast Cancer

**DOI:** 10.1155/2019/2183057

**Published:** 2019-11-21

**Authors:** Rui-min Ma, Fan Yang, Du-ping Huang, Min Zheng, Yi-luan Wang

**Affiliations:** ^1^Department of Breast Surgery, The Second Affiliated Hospital and Yuying Children's Hospital of Wenzhou Medical University, Zhejiang, Wenzhou, China; ^2^Department of Breast and Thyroid Surgery, The First Affiliated Hospital of Wenzhou Medical University, Zhejiang, Wenzhou, China; ^3^Department of Anesthesiology, The Second Affiliated Hospital and Yuying Children's Hospital of Wenzhou Medical University, Zhejiang, Wenzhou, China

## Abstract

**Aim:**

To investigate the mRNA expression and clinical significance of structural maintenance of chromosomes protein 4 (*SMC4*) in breast cancer.

**Methods:**

A total of 23 paired samples were sequenced, and data from the Cancer Genome Atlas were analyzed.

**Results:**

*SMC4* mRNA level was significantly upregulated in breast cancer tissues (*P* < 0.001). Patients with high mRNA expression of *SMC4* had significantly poor survival (*P* = 0.012). Subgroup analyses show that in nontriple negative breast cancer (non-TNBC) patients, the high *SMC4* mRNA expression, older age (>65), negative progesterone receptor, and advanced stages (III-IV) were independent risk factors (HR = 3.293, 95% CI 1.257-8.625, *P* = 0.015). In patients with TNBC, high mRNA expression of *SMC4* correlated with better survival rate (*P* < 0.046).

**Conclusion:**

*SMC4* mRNA level is a good prognostic biomarker for patients with breast cancer.

## 1. Introduction

Breast cancer is the most prevalent cancer among women globally. Recent data shows that about 2,080,000 new cases of breast cancer are diagnosed yearly, while more than 626,000 patients are dying of this disease each year [1]. The evolution of molecular subtype classification has incentivized the design of more precise and effective therapies for patients. Currently, it has been reported that early surgical treatment of breast cancer leads to good prognostic outcomes. However, about 10% of patients experience metastasis before death within 5 years after surgery [2–4]. Therefore, identification of new biomarkers is needed to facilitate the development of more effective therapeutic strategies.

Structural maintenance of chromosomes protein 4 (SMC4) is encoded by *SMC4*, which located in 3q25.33 (https://www.ncbi.nlm.nih.gov/gene/10051). SMC4 is a member of the SMC family. This ATPase family maintains the stability of chromosomal structure and participates in mitosis of eukaryotic cells [5, 6]. It has been shown that condensin, a heterodimer composed of SMC4 and SMC2, is involved in chromatin condensation and gene regulation [5, 7]. Other researchers have found a relationship between *SMC4* and tumors. Zhou et al. discovered that *SMC4* is highly impressed in human primary hepatocellular carcinoma [8]. High expression of *SMC4* is an independent predictor of poor survival in patients with colorectal cancer [9, 10]. Recently, Jiang et al. found that the high expression of *SMC4* is related to the aggressiveness of glioma [11]. However, the mechanisms have not been resolved.

In patients with breast cancer, *SMC4* is thought to be one of the 28 genes related to paclitaxel resistance [12]. It has also been suggested that *SMC4* contributes to the risk of distant metastasis of lymph node-negative primary breast cancer [13]. Nevertheless, the succinct role of *SMC4* in breast cancer and the relationship between *SMC4* expression and clinical outcomes remain unknown.

In this study, we analyzed the expression of *SMC4* in breast cancer tissues and adjacent noncancerous tissue. We further explored the clinical value of *SMC4* expression.

## 2. Materials and Methods

### 2.1. Patient Specimen

A total of 23 breast cancer tissues and 23 paired adjacent noncancerous tissues were obtained from patients in the First Affiliated Hospital of Wenzhou Medical University. The tissues were harvested during surgery and immediately snap-frozen in liquid nitrogen. Total RNA was then extracted from the tissue samples using TRIzol agent (Life Technologies, California, USA). The complementary DNA (cDNA) libraries were prepared for single-end sequencing using Ion Total RNA-Seq Kit v2.0 (Life Technologies). The cDNA libraries were then processed for the Proton (Life Technologies) sequencing process. Data acquisition and patient enrollment were approved by the clinical ethics committee of the First Affiliated Hospital of Wenzhou Medical University.

### 2.2. Retrieval of Publicly Available Data

Public data of *SMC4* gene expression and clinicopathological characteristics for patients with breast cancer were obtained from the Cancer Genome Atlas (TCGA) database. The expression of human epidermal growth factor receptor 2 (HER-2) determined by immunohistochemical assay was further adjusted to the fluorescence of in situ hybridization assays. A total of 992 female patients enrolled from 2001 to 2013. Among them, 113 patients had paired cancerous and normal sample data.

### 2.3. Statistical Analysis

Paired continuous variables were analyzed by paired *t*-test. Categorical variables were expressed as number of cases (percentages) and analyzed using the *χ*^2^ test. Survival analyses were performed by Kaplan-Meier survival analysis and multivariable Cox regression analysis, along with hazard rate (HR) and 95% confidence interval (95% CI). SPSS version 22.0 (SPSS Inc., Chicago, IL, USA) and GraphPad Prism version 7.0 (GraphPad Software Inc., La Jolla, CA, USA) were used for data analyses. A two-tailed *P* < 0.05 was considered significantly different.

## 3. Results

### 3.1. SMC4 Is Upregulated in Breast Cancerous Tissue

**Figure 1:**
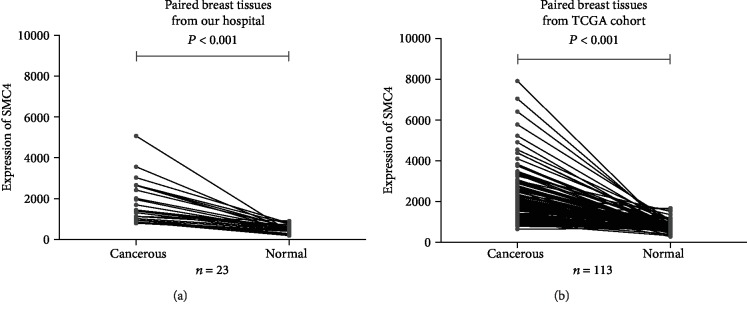
The mRNA expression levels of SMC4 upgraded in cancerous tissues, both in the 23 paired human specimens (a) (*P* < 0.001) and in the TCGA dataset (b) (*P* < 0.001).

Results showed that *SMC4* mRNA expression was higher in all the 23 breast cancerous tissues than in all the 23 paired adjacent noncancerous tissues (Figure 1(a)). In most of the breast cancerous tissues (18/23), the expression was 2-fold higher compared to the paired nonbreast cancer tissues. The log2 fold change was about 1.75. In the TCGA data, *SMC4* mRNA expression was similarly higher in cancerous tissues than in paired noncancerous tissues (*P* < 0.001) (Figure 1(b)).

### 3.2. Relationship between SMC4 mRNA Expression and Clinicopathological Characteristics

In TCGA cohort, 992 female patients were enrolled from 2001 to 2013. As shown in Table 1, there were no significant differences in age, menopause, lymph node metastasis, distant metastasis, and HER-2 state, between the high *SMC4* mRNA expression group and the low *SMC4* mRNA expression group (all *P* values > 0.05). However, there were significant differences between the two groups when adjusted to tumor categories, ER state or PR state (all *P* values < 0.05).

### 3.3. Survival Analyses

**Figure 2:**
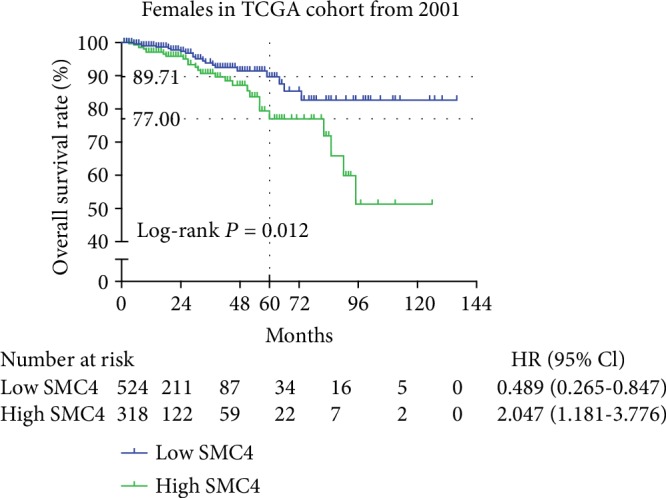
Kaplan-Meier analysis indicated that breast cancer patients with higher mRNA expression of SMC4 had worse survival in TCGA dataset (*P* = 0.012).

As shown in Figure 2, the 5-year overall survival (OS) rate of the low *SMC4* group was 89.71%, whereas the OS rate of the high *SMC4* group was 77.00%, indicating a significantly lower OS rate in the high *SMC4* group than in the low *SMC4* group (*P* = 0.012).

**Figure 3:**
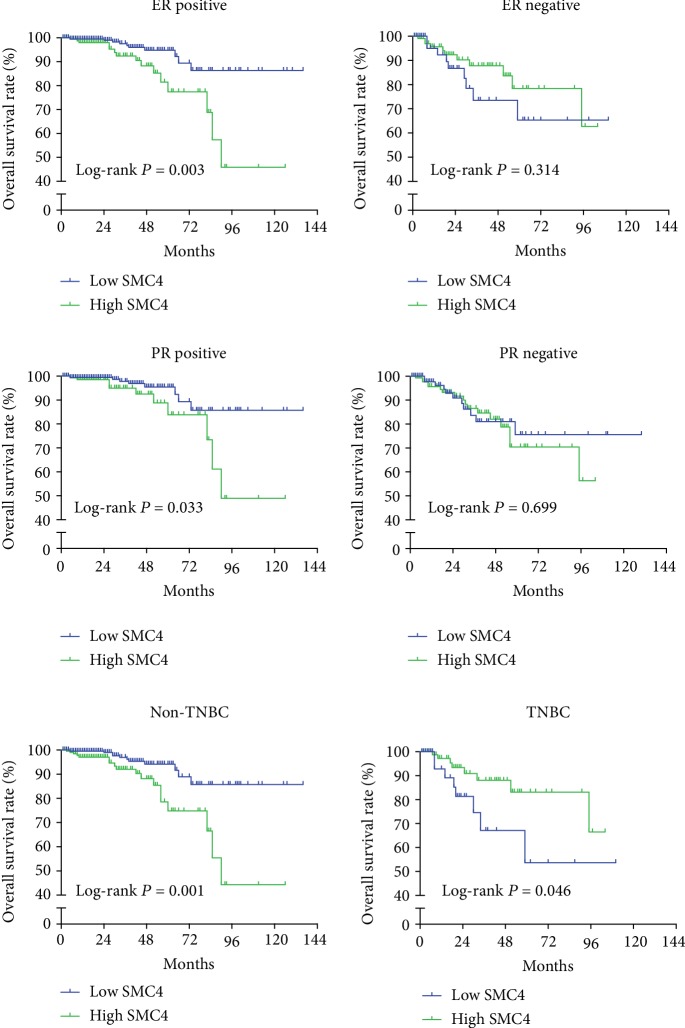
Subgroup analyses indicated that in patients with ER positive or PR positive or nontriple negative breast cancer (non-TNBC), higher mRNA expression of SMC4 was associated with lower survival rate (*P* = 0.003, *P* = 0.033, and *P* = 0.001, respectively). In patients with TNBC, higher mRNA expression of SMC4 correlated with higher survival rate (*P* = 0.046). There was no significant difference in ER/PR-negative patients between the higher SMC4 mRNA expression group and the lower SMC4 mRNA expression group.

Further subgroup analyses revealed that the OS rates were markedly lower in high *SMC4* expression groups in patients with ER positive or PR positive (*P* = 0.003 and *P* = 0.033, respectively) (Figure 3). Nevertheless, there were no significant differences about OS rates in patients with ER negative or PR negative (all *P* > 0.05) (Figure 3). In patients with nontriple negative breast cancer (non-TNBC), the high *SMC4* expression group had poor prognosis (*P* = 0.001) (Figure 3). However, among the patients with TNBC, the high *SMC4* expression group had better outcomes (*P* = 0.046) (Figure 3).

**Figure 4:**
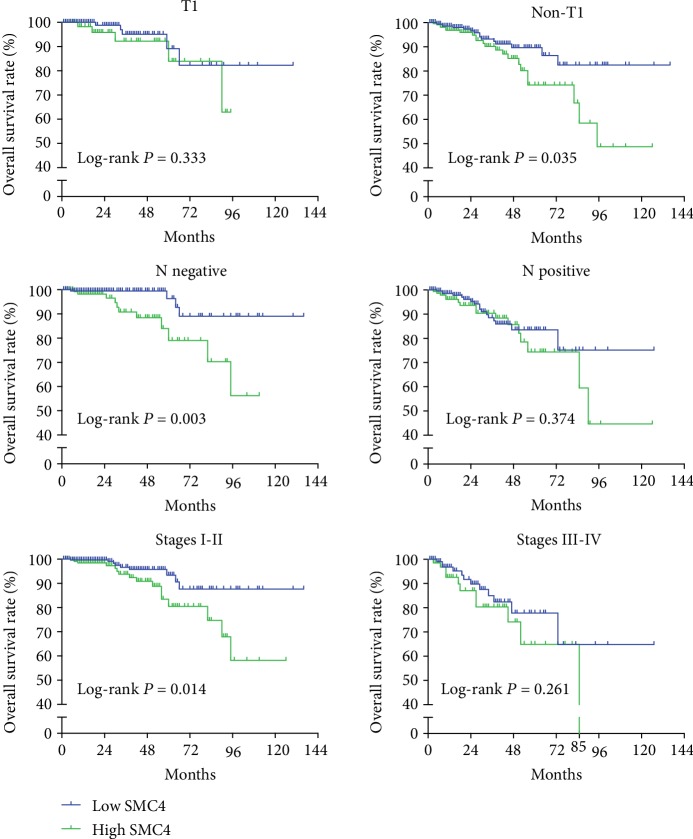
Subgroup analyses indicated that in breast cancer patients with larger tumors (>T1), without lymph node metastasis, or stages I-II, higher mRNA expression of SMC4 was associated with low survival rate (*P* = 0.035, *P* = 0.003, and *P* = 0.014, respectively). In patients with T1, lymph node metastasis, or stages III-IV, there was no significant difference in T1, lymph node metastasis, or stages III-IV, between the higher SMC4 mRNA expression group and the lower SMC4 mRNA expression group.

Figure 4 shows that there was no significant difference between the high SMC4 mRNA expression group and the low *SMC4* mRNA expression group in terms of tumor category 1 (T1), lymph node (N) metastases, or stages III-IV (all *P* > 0.05). However, other subgroups according to non-T1, lymph node negative, or stages I-II showed positive results that high *SMC4* expression groups had significantly lower OS rates (*P* = 0.035, *P* = 0.003, and *P* = 0.014, respectively).

As shown in Table 2, when adjusted to non-TNBC, age, stage, PR state, and the *SMC4* mRNA expression were found to be significant independent risk factors of prognosis (hazard ratio (HR) = 7.372, 95% confidence interval (CI) 2.902-18.727, *P* < 0.001; HR = 4.370, 95% CI 1.745-10.949, *P* = 0.002; HR = 4.296, 95% CI 1.628-11.336, *P* = 0.003; and HR = 3.293, 95% CI 1.257-8.625, *P* = 0.015, respectively).

## 4. Discussion

Breast cancer is the most prevalent malignance in women. Since 1989, it was reported that the mortality rate due to this cancer has been decreasing over the years. However, this trend has slowed down [14]. Therefore, new strategies are needed.

*SMC4* had been found highly expressed in several cancers, such as glioma, colorectal carcinoma, and hepatocellular carcinoma [8, 9, 11]. 3q25, the locus of SMC4 belonging to, had been proved to be involved in high level of recurrent DNA amplifications in breast cancer cell lines [15, 16]. Here, we show that the mRNA expression of *SMC4* was upregulated in invasive breast cancer cells. Upregulated *SMC4* mRNA level could improve the sensitivity of Cdk1 to drive chromatin compaction at mitotic entry [17] and increase the aggressiveness, proliferation, and dedifferentiation of cancer cells [11, 18]. This may explain why larger tumors have higher *SMC4* mRNA expression in our study. Moreover, the high expression of *SMC4* may increase double-stranded DNA breaks by enhancing the action of topoisomerase II [19] and cause mutations and mismatches resulting in unique chromosomal rearrangements in breast epithelial cells [20]. And it has been reported that overexpression of *SMC4* activates JAK2/Stat3 and TGF*β*/Smad pathway and promotes aggressiveness of cancer cells [8, 11].

Previous studies reported that in ER-positive breast cancer, PLK1 increased ER transcriptional activity and ER expression and then induced the invasion [21, 22]. PLK1 could be upregulated by the high expression of SMC4 [23]. That may be the reason why high *SMC4* expression levels were associated with worse survival in ER/PR-positive patients in the present study. And in HER-2-positive breast cancer, PLK1-siRNA suppresses cancer growth and metastasis [24]. In the present study, there was a trend that HER-2-positive patients with high mRNA expression of *SMC4* may suffer lower survival rate (Supplementary Fig. ([Supplementary-material supplementary-material-1])). These findings indicate that overexpression of SMC4 may lead to cancer progression and poor prognosis through PLK1 in non-TNBC. Together, our results suggest that SMC4 has the potential to be an independent prognostic predictor and therapeutic target in non-TNBC.

However, in patients with TNBC, overexpression of *SMC4* mRNA correlated with better survival. It may be related to SMC4 modulating the sensitivity of breast cancer cells to paclitaxel treatment [12]. Moreover, *SMC4* overexpression triggers the formation of double-stranded DNA breaks and unique chromosomal rearrangements, leading to impaired DNA mismatch repair [19, 20]. While DNA mismatch repair plays a central role in the development of drug resistance in TNBC cancer cells, the high expression of *SMC4* may lead to long-term effect of chemotherapy.

Additionally, we found that in T2-3N0 or ER/PR-positive patients, higher mRNA expression level of *SMC4* was associated with worse survival rates. This implies that the mRNA expression of *SMC4* would be a useful tool to identify patients who need more aggressive therapy and those with low relapse risk.

In conclusion, we found that mRNA expression of *SMC4* was upregulated in invasive breast cancer cells. Furthermore, patients with high mRNA level of *SMC4* suffered different survival with TNBC and non-TNBC. And SMC4 could be a good biomarker for predicting the prognosis and potential for therapeutic target.

## Figures and Tables

**Table 1:** The relationship between SMC4 expression and clinicopathological characteristics in female breast cancer patients from the TCGA cohort enrolled from 2001 to 2013 (*n* = 992).

Clinicopathological characteristic	Low SMC4 (%) (*n* = 614)	High SMC4 (%) (*n* = 378)	*χ* ^2^	*P*
Age			2.465	0.117
≤65	428 (60.4%)	281 (39.6%)		
>65	186 (65.7%)	97 (34.3%)		
Menopause			2.165	0.141
Premenopause	146 (57.5%)	108 (42.5%)		
Postmenopause	387 (62.8%)	229 (37.2%)		
Tumor categories			8.993	0.003
T1	181 (69.6%)	79 (30.4%)		
T2-T4	432 (59.1%)	299 (40.9%)		
Lymph node metastasis			1.359	0.244
None	289 (59.8%)	194 (40.2%)		
Present	166 (31.6%)	227 (18.2%)		
Distant metastasis			0.077	0.781
No	489 (59.1%)	339 (40.9%)		
Yes	10 (62.5%)	6 (37.5%)		
Clinical stage			1.954	0.162
I-II	449 (60.7%)	291 (39.3%)		
III-IV	159 (65.7%)	83 (34.3%)		
Estrogen receptor			71.256	<0.001
Negative	80 (37.4%)	134 (62.6%)		
Positive	508 (69.2%)	226 (30.8%)		
Progesterone receptor			58.413	<0.001
Negative	141 (44.9%)	173 (55.1%)		
Positive	445 (70.5%)	186 (29.5%)		
HER-2			0.036	0.849
Negative	457 (62.1%)	279 (37.9%)		
Positive	111 (61.3%)	70 (38.7%)		
TNBC			72.197	<0.001
Yes	50 (32.1%)	106 (67.9%)		
No	517 (68.3%)	240 (31.7%)		

HER-2 = human epidermal growth factor receptor 2; TNBC = triple negative breast cancer; SMC4 = structural maintenance of chromosomes protein 4 mRNA.

**Table 2:** Multivariate Cox regression analysis for risk factors associated with OS in female non-TNBC patients from the TCGA cohort from 2001 to 2013 (*n* = 992).

Characteristics	HR	95% CI	*P*
Age >65 vs. ≤65	7.372	2.902-18.727	<0.001
Stages III-IV vs. stages I-II	4.370	1.745-10.949	0.002
PR negative vs. PR positive	4.296	1.628-11.336	0.003
High SMC4 vs. low SMC4	3.293	1.257-8.625	0.015

TNBC = triple negative breast cancer; HR = hazard ratio; CI = confidence intervals; PR = progesterone receptor; SMC4 = structural maintenance of chromosomes protein 4. Wald forward with SMC4 expression and all the characteristics.

## Data Availability

The data used to support the findings of this study are included within the supplementary materials.

## Supplementary Materials

Supplementary Materials There was only a trend that HER-2-positive patients with high mRNA expression of SMC4 may suffer lower survival rate whether ER/PR positive or not (all *P* > 0.05).
